# Genetic and Non Genetic Hearing Loss and Associated Disabilities: An Epidemiological Survey in Emilia-Romagna Region

**DOI:** 10.3390/audiolres11030043

**Published:** 2021-09-16

**Authors:** Elisabetta Genovese, Silvia Palma, Valeria Polizzi, Giovanni Bianchin, Michela Cappai, Shaniko Kaleci, Alessandro Martini, Andrea Ciorba, Paolo Stagi

**Affiliations:** 1 Audiology, Department of Diagnostic, Clinical and Public Health Medicine, University of Modena and Reggio Emilia, 41100 Modena, Italy; Elisabetta.genovese@unimore.it; 2 Audiology, Primary Care Unit, 41100 Modena, Italy; 3 Department of Audiology, Santa Maria Nuova Hospital, Center for Clinical and Basic Research (IRCCS), Reggio Emilia, 71013 San Giovanni Rotondo, Italy; valeria.polizzi@ausl.re.it (V.P.); giovanni.bianchin@ausl.re.it (G.B.); 4 Refer for Children and Adolescent Mental Health Services, Territorial Assistance Service, Emilia Romagna Region, 40127 Bologna, Italy; michela.cappai@regione.emilia-romagna.it; 5 Department of Surgical, Medical, Dental and Morphological Sciences with Interest in Transplant, Oncological and Regenerative Medicine, University of Modena and Reggio Emilia, 41100 Modena, Italy; shaniko.kaleci@ausl.bologna; 6 Interdepartmental Research Centre “I–APPROVE—International Auditory Processing Project in Venice”, University of Padua, Santi Giovanni e Paolo Hospital, ULSS3 Serenissima, 30100 Venice, Italy; alessandromartini@unipd.it; 7 ENT & Audiology Unit, Department of Neurosciences, University Hospital of Ferrara, Via Aldo Moro, 8, 44124 Cona, Italy; andrea.ciorba@unife.it; 8 Mental Health Department, AUSL Toscana Centro, 50053 Empoli, Italy; paolo.stagi@uslcentro.toscana.it

**Keywords:** hearing loss, hearing screening, disabilities, epidemiology, Emilia Romagna region, Italy

## Abstract

Hearing loss is one of the most common congenital sensory disorders. It can be associated with several comorbidities, in particular developmental disabilities (DD). In Emilia-Romagna (ER), a region in Northern Italy, Child and Adolescent Mental Health Services (CAMHS) provide the diagnostic framework and treatment for these conditions. The aim of the present study is to evaluate the prevalence of hearing loss, both isolated or in association with comorbidities, in the juvenile population. The study draws its data from the ER Childhood and Adolescent Neuropsychiatry Information System (SINPIAER), an Administrative Healthcare Database collecting the clinical data of all those who have attended CAMHS since 2010. The most frequent type of hearing loss was bilateral sensorineural hearing loss, which was present in 69–72% of the cases, while bilateral conductive hearing loss was the second most common type, ranging from 8 to 10%. Among DD, congenital malformations, mental retardation, visual impairment, and cerebral palsy were the most common. In particular, autism spectrum disorders show increasing incidence and prevalence among CAMHS users in ER region. In-depth knowledge of hearing loss epidemiology and related conditions, such as developmental disabilities, in the juvenile population is crucial for disease prevention, health planning, and resource allocation.

## 1. Introduction

Hearing loss is one of the most common congenital sensory disorders, affecting one out of every 3000 newborns [1,2]. An early diagnosis and intervention are important for the acquisition of speech and linguistic abilities, and for this reason, the Joint Committee on Infant Hearing (JCIH) has recommended the use of protocols to facilitate hearing screening and intervention programs [3].

Since January 2012, Emilia Romagna (ER), a region of nearly 4.5 million people in northern Italy, has extended the newborn hearing screening (NHS) program to all of its counties and has contextually endorsed a hearing disabilities multidisciplinary regional group that includes many professionals such as neonatologists, audiologists-otolaryngologists, infantile and adolescence neuropsychiatrists, and speech therapists [4]. The group has the role of facilitating technical functions and coordination among the professionals and operating units involved in this pathway.

Child and Adolescent Mental Health Services (CAMHS) are spread over the local territory on the basis of geographical area and the number of inhabitants and represent an important resource out of the hospital, providing a diagnostic framework and clinical monitoring. 

They deal with developmental disabilities (DD) that represent a group of chronic conditions that may result in functional limitations and that often require life-long support [5,6], providing treatments such as speech therapy, physiotherapy, and interventions dedicated to a child’s integration at school. 

Trends in the prevalence of DDs and their co-occurrence need to be measured to plan community and medical resources. In particular, children with significant disabilities and hearing loss face a great set of challenges [7] since about 30–40% of children receiving cochlear implants also have other comorbidities, as previously reported [8,9,10]. 

Updated and improved estimates of children and adolescents with disabilities are important to better quantify the burden of disease and the resources required to address the needs and rights of these children [11]. 

This study is a regional survey based on the evaluation of CAMHS data and aims to deduce the prevalence of hearing loss in the juvenile population that is associated with other DDs. The analysis was focused on congenital malformations, visual impairment, mental retardation, cerebral palsy, and autism spectrum disorders (ADS). In particular, the overall prevalence and trends over time have been considered.

## 2. Materials and Methods

The Italian National Health System provides universal coverage and is organized at three levels: national, regional, and local. Administrative data are collected by local health units and are processed at the regional level.

The analysis involves data of an IT management program called Sinpiaer, which used by all the regional CAMHS, and was fully introduced in 2011, with the aim of monitoring the implementation and analysis of intervention programs.

Sinpiaer can also be accessed by those aged < 18 years old who under the care of CAMHS before their transfer to adult healthcare/social services. 

The program generates an annual data flow, reporting personal data, clinical features, and the date and number of health care treatments received by CAMHS users. Clinical conditions are classified according to the Manual of International Statistical Classification of Diseases and Related Health Problems, ICD 10th revision, as for all clinical conditions [12]. 

The present study was performed using the IC10 codes for hearing loss (H.90–H.91):

H90 Conductive and sensorineural hearing loss:
H90.0 Conductive hearing loss, bilateral;
H90.1 Conductive hearing loss, unilateral with unrestricted hearing on the contralateral side;
H90.2 Conductive hearing loss, unspecified;
H90.3 Sensorineural hearing loss, bilateral;
H90.4 Sensorineural hearing loss, unilateral with unrestricted hearing on the contralateral side;
H90.5 Sensorineural hearing loss, unspecified;
H90.6 Mixed conductive and sensorineural hearing loss, bilateral;
H90.7 Mixed conductive and sensorineural hearing loss, unilateral with unrestricted hearing on the contralateral side;
H90.8 Mixed conductive and sensorineural hearing loss, unspecified.

H91 Other hearing loss:
H91.8 Other specified hearing loss;
H91.9 Hearing loss, unspecified.

Code H91.3, Deaf mutism, not elsewhere classified, was excluded from the analysis, as no cases were present in the Simpiaer database).

Code attribution to clinical conditions was made by neuropsychiatrists from the services that attended a specific training course on the ICD 10 system. The same professionals inserted the DC into the IT program. Concerning hearing loss, DC was attributed based on audiologists/ otorhinolaryngologists reports.

Data collection was performed including data from 1 January 2011 to 31 December 2020, and the extrapolated data were:

- Prevalence and incidence of H90–H91 users among the general population and among CAMHS users;
- Gender and citizenship of H90–H91;
- Distribution by age range: 0–2 years, 3–5 years, 6–10 years, 11–13 years,14–17 years, and 18 years. Age range groups are related to school-aged children: maternal school, primary school, secondary school;
- Number and percentage of users with isolated diagnostic codes (DC) H90-H91;
- Number of DC H90-H91 with the following other associated codes:
  Q00–Q99 Congenital malformations, deformations and chromosomal abnormalities;
  Q10–Q18 Congenital malformations of eye, ear, face, and neck;
  Q35–Q37 Cleft lip and cleft palate;
  Q67 Congenitalmusculoskeletal deformities of head, face, spine, and chest;
  Q75 Other congenital malformations of skull and face bones;
  Q80–Q89 Other congenital malformations;
  Q90–Q99 Chromosomal abnormalities not elsewhere classified;
  F70–F79 Mental retardation;
  F84 Pervasive developmental disorders (autism spectrum disorders listed here);
  G80–G83 Cerebral palsy and other paralytic syndromes;
  H3–H59 Visual impairment;
  P00–P96 Certain conditions originating in the perinatal period.

The Q00–Q99 Group, including congenital malformations, deformations, and chromosomal abnormalities was analysed by age, and its prevalence in the general juvenile population was calculated. 

This is an observational retrospective chart review study; the study did not affect patient care in any way since the study only incorporates the recordings of a database and its evaluation. It was conducted according to the guidelines of the declaration of Helsinki. 

## 3. Results

The prevalence and incidence of hearing loss in the juvenile population under the care of regional CAMHS are represented in Table 1: the prevalence of the population referred to CAMHS increased slightly over the years (except in 2020, year of the COVID emergency), while the percentage of H90–H91 codes was stable for the entire period. 

**Figure 1:**
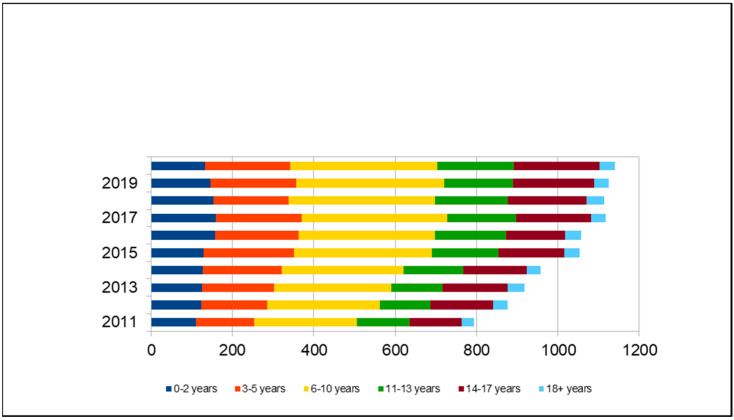
Distribution of ICD-10 codes H90-H91 by age group.

The cohort of H90–H91 subjects is represented by 58% males and 48% females; 23% are foreign citizens. The age range distribution is indicated in Figure 1, in which a greater representation of the 6–10 years group is evident.

**Figure 2:**
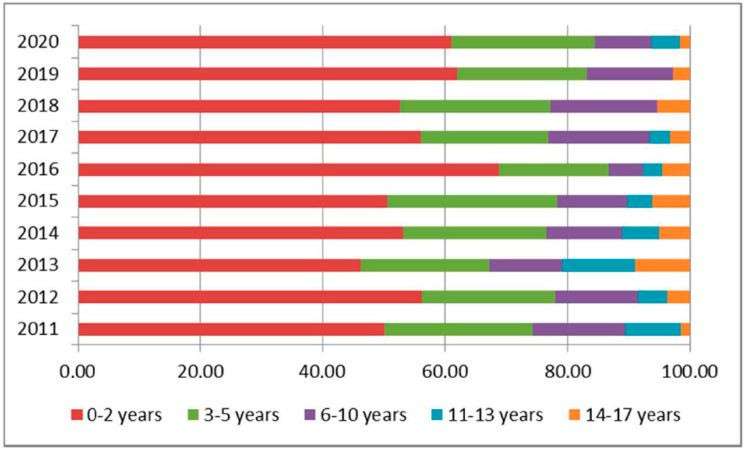
Proportion of hearing loss by age.

**Figure 3:**
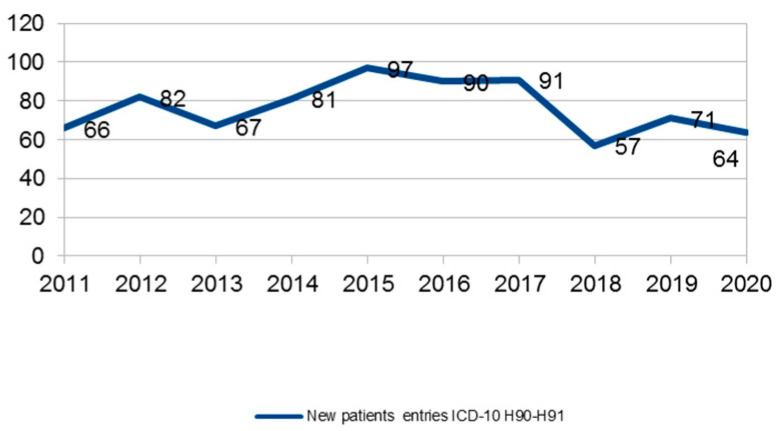
New diagnosis at CAMHS (codes H90–H91) per year.

Those aged 0–2 years are mainly identified through newborn hearing screening (NHS), and this group has a stable incidence, representing 12–14% of the population in charge and 50–60% of new cases under the care of CAMHS each year (Figure 2, Table 2). A graphic demonstrating the trend of new diagnoses per year is represented in Figure 3.

**Figure 4:**
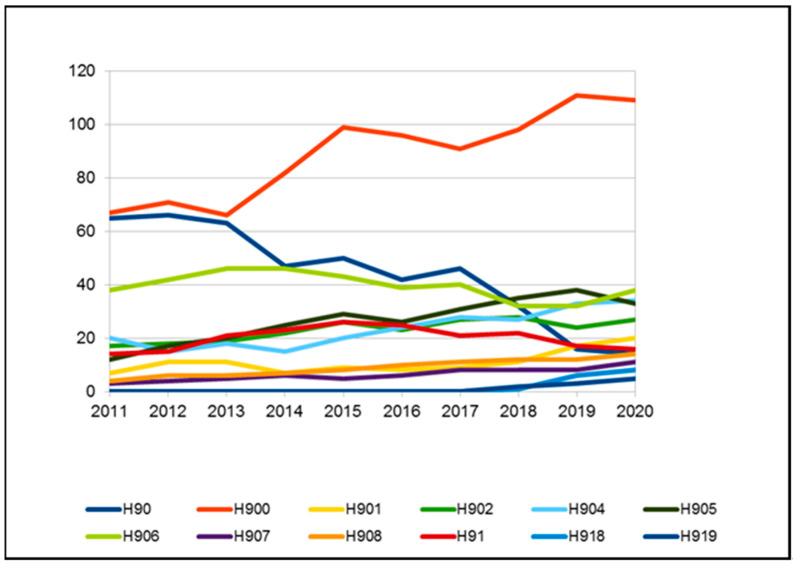
Subjects affected by hearing loss subdivided according IC10 classification into further H90 subtypes.

Subjects affected by hearing loss, subdivided according to ICD 10th classification into further subtypes H90–H91, are indicated in Table 3 and in Figure 4. The most frequent type of hearing loss was bilateral sensorineural hearing loss (code H90.3), which occurred in 69–72% of cases, while bilateral conductive hearing loss (H90.2) was the second most common type, ranging from 8 to 10%. Over the years, the use of the generic code H90, without further specification, dropped drastically, from 8% to 1%. 

**Figure 5:**
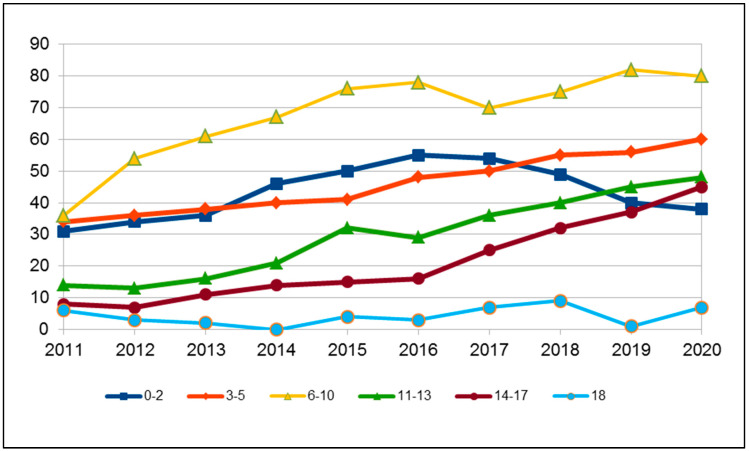
Q code user distribution by age range.

Children admitted to CAMHS with just a hearing loss code (H90–H91) progressively decreased from 58% in 2011 to 40% in 2020 (Figure 5). Inversely, cases with other associated codes (therefore with multiple disabilities) increased over the years (Table 4).

Concerning DDs, congenital malformations, mental retardation, visual impairment, and cerebral palsy were the most frequent; in particular, autism spectrum disorders (F84 code) show growing incidence and prevalence among CAMHS users in the ER region; P codes slightly decreased over the years (Table 5) 

Genetic malformations and genetic syndromes (Q codes), with a percentage comprised between 13.9–17.1%, represent the most frequent condition. The percentage of children with code Q has progressively increased from 16% to 24% in last five years (calculated by the number of Q cases in relation to H90–H91 cases). Prevalence has increased constantly (Table 6). 

Going into detail, Q codes increased in children older than 6 years of age, while they were stable in very young children (Figure 5). 

## 4. Discussion

When planning and allocating resources for hearing disability intervention programs, epidemiological data are crucial. 

The present study demonstrates significant data on regional hearing loss prevalence and related conditions in the young population accessing CAMHS, and, to our knowledge, this is one of the first investigations of a regional database with this purpose in Italy. Hearing loss is a complex condition that can occur at any age and is associated with a wide array of etiologies [13,14]. In the ER cohort, the prevalence of subjects receiving health care in the public system for hearing loss was about 1–1.6:1000, substantially similar to that of the general estimates from the neonatal population [1,2] This percentage may be underestimated, as some children affected by mild and moderate hearing loss might be not under the care of CAMHS. The worldwide prevalence rates of hearing loss in children are becoming more and more available nowadays, mainly because of the implementation of NHS programs, but much of the existing data concern older children [15]. 

The prevalence of hearing loss in the juvenile population, which is stratified by age, is important, as it enables a better identification of those cases at late onset, not identifiable at the NHS, and more detailed epidemiological data can direct efforts to prevent its progression [16]. It has been estimated that the majority of children with disabilities live in low and middle-income countries [17]. Before the introduction of NHS programs, on average, children usually received a diagnosis of hearing loss at the age of 2 average [18]. The analysis of the distribution of H90-H91 users has confirmed the benefits of NHS programs, as 50–60% of new hearing loss cases per year are diagnosed in the first 2 years of life.

In the literature, calculating prevalence reflects the use of different criteria to include and describe cases with hearing impairment, according to type, entity, and age of children. 

In this regard, the estimated prevalence of hearing loss per 1000 children was 6.4 in a 2009–2016 National Health Interview Survey concerning children aged 3–17 years [19] based on a sample of children randomly selected and through in-person, computer-assisted interviews with a knowledgeable parent. 

There is a need for uniformity in collecting data to determine the real prevalence and incidence of hearing loss cases. 

The number of generic H90 codes has decreased, confirming an improvement in the appropriate use of the ICD 10 classification system. The majority of cases was due to permanent hearing impairment, which is caused by both genetic and environmental factors, and it is estimated that a genetic factor is responsible for about 50% of all congenital sensorineural hearing loss cases [20,21,22]. Much of the prevalence of pediatric hearing loss is due to acquired etiologies, but specific contributions to that global prevalence have not been well documented [23].

A growing number of unilateral hearing loss cases, underestimated in the past years, was found. This demonstrates the prompt response of the public health services to the identification of different hearing loss types, as the diagnosis and management of pediatric hearing loss have changed significantly in recent years. The main reasons are the implementation and diffusion of NHS and the development of a new generation hearing aids and cochlear implants. 

DDs due to an impairment of physical, learning, language, or behavior abilities usually require personalized programs. A recent review reveals that the number of children and adolescents with disabilities in the world is at least 291 million globally and that this prevalence increases with age [11].

Trends in the prevalence of DD may change due to risk factors or to preventative public health measures. In particular, improvements in maternal and child health care and an increased community awareness of disorders may contribute to improving the diagnosis of different DD.

The WHO’s Global Burden of Disease Study (GBD) estimated that in 2004, at least 93 million children and adolescents (0–15 years) worldwide (5.1% of the global total) lived with a moderate-to-severe disability and that 13 million (0.7%) had a severe disability [17]. These estimates were generated from four specific impairments: epilepsy, intellectual disability, hearing loss, and vision loss. 

A study from the USA recently reported that the percentage of children (age 3–17 years) diagnosed with a developmental disability has increased, resulting in a growing population of children with one or more developmental disabilities [24]. 

In the ER cohort, the percentage of H90–H91 users with other associated DDs progressively increased from 42% to 60% in recent years, which is probably due to different factors: syndrome diagnosis and treatment improvements besides awareness in the family of therapeutic possibilities. On the contrary, the improved neonatal survival rate, particularly among low birth weight and preterm infants [25] does not substantially interfere, as P code percentages have decreased over the years

The Q code group, which describes congenital malformations and chromosomal abnormalities, was the condition that was the most frequently associated with hearing loss. More than 400 syndromes associated with hearing loss have been described, and syndromes represent about 30% of all genetic causes, which are often associated with a delayed onset of hearing loss [26]. Prevalence calculated on the general juvenile population demonstrates a slow but steady increase in a relatively short time (ten years), which is partly due to the improvement in the appropriate use of the ICD system by professionals, and this finding will be the subject of further study.

Cerebral palsy, a heterogeneous condition in terms of etiology and severity of impairments, is the most common childhood physical disability [27]. This study presented a reduced prevalence over the years, from 5.5% to 3.6%, which is very similar to Dufresne’s report that documented a hearing impairment in 12.7% of cases, with 2.7% overall having a severe hearing loss [28]. 

A stable percentage of children presented both visual and hearing impairment. The H3-H5 codes include a wide variety of clinical conditions; in any case, these children, who are facing two sensorial impairments, need personalized high-intervention programs, using many rehabilitative service resources.

The percentage of F84 codes ranged from 1.3 to 2.4% in the studied population, indicating the importance of the audiological evaluation in these subjects in order to rule out the presence of hearing problems in those subjects.

The major strengths of this study are its large sample size and its regional dimension, allowing the analysis of children with developmental disabilities associated with hearing loss and regarding the use of the CAMSH by these children. 

Major limitations are the relatively short observation time and the analysis by DC that does not allow us to evaluate the entity of hearing loss of CAMHS users.

## 5. Conclusions

Knowing the hearing loss prevalence of the juvenile population is important when planning and allocating resources for the governance of health services. 

The most frequent type of hearing loss was bilateral sensorineural hearing loss, while a growing number of unilateral hearing loss cases, which have been underestimated in past years, was found.

In the ER cohort, the percentage of H90–H91 users with other associated DDs progressively increased from 42% to 60% in recent years, and congenital malformations and chromosomal abnormalities have resulted the conditions that are the most frequently associate with hearing loss. Autism spectrum disorders show increasing incidence and prevalence.

Evaluating the prevalence of DD is crucial for planning educational, medical, and community resources, as an increasing trend has been confirmed in this regional survey.

The results of this study confirmed the importance of a robust IT management for the governance of health service resources. 

## Figures and Tables

**Table 1:** Prevalence and incidence of hearing loss in the juvenile population and in CAMHS users. Due to small numbers, incidence was measured as 1:10,000 instead of 1:1000.

Years	Pop. Age 0–17	Patients under UONPIA-ER Care	Patients H.90–91 Diagnosis under UONPIA-ER Care	Prevalence:1000	Incidence:10.000
**2011**	695.043	41.012	794 (1.9%)	1.1	0.95
**2012**	704.716	45.079	876 (1.9%)	1.2	1.16
**2013**	711.268	48.486	917 (1.9%)	1.3	0.94
**2014**	712.298	50.660	957 (1.9%)	1.3	1.14
**2015**	714.051	52.132	1053 (2%)	1.5	1.36
**2016**	713.391	54.007	1058 (2%)	1.5	1.26
**2017**	711.765	57.996	1118 (1.9%)	1.6	1.28
**2018**	708.622	59.897	1114 (1.9%)	1.6	0.80
**2019**	704.439	62.353	1125 (1.8%)	1.6	1.01
**2020**	698.003	56.405	1140 (2%)	1.6	0.92

**Table 2:** Incidence of hearing loss by age in percentage.

Age Group						
**Year**	**0–2 Years**	**3–5 Years**	**6–10 Years**	**11–13 Years**	**14–17 Years**	**Total**
2011	50.00	24.24	15.15	9.09	1.52	100
2012	56.10	21.95	13.41	4.88	3.66	100
2013	46.27	20.90	11.94	11.94	8.96	100
2014	53.09	23.46	12.35	6.17	4.94	100
2015	50.52	27.84	11.34	4.12	6.19	100
2016	68.89	17.78	5.56	3.33	4.44	100
2017	56.04	20.88	16.48	3.30	3.30	100
2018	52.63	24.56	17.54	0.00	5.26	100
2019	61.97	21.13	14.08	0.00	2.82	100
2020	60.94	23.44	9.38	4.69	1.56	100

**Table 3:** Subjects (in percentage) affected by hearing loss, subdivided according IC10 classification into further H90 subtypes.

ICD-10 Diagnosis	2011	2012	2013	2014	2015	2016	2017	2018	2019	2020
**H90**	8.19	7.53	6.87	4.91	4.75	3.97	4.11	2.87	1.42	1.23
**H900**	8.44	8.11	7.20	8.57	9.40	9.07	8.14	8.80	9.87	9.56
**H901**	0.88	1.26	1.20	0.73	0.85	0.76	0.81	0.99	1.51	1.75
**H902**	2.14	2.05	2.07	2.30	2.47	2.17	2.42	2.51	2.13	2.37
**H903**	**68.89**	**69.75**	**70.01**	**70.74**	**70.09**	**71.74**	**72.09**	**72.35**	**71.82**	**71.14**
**H904**	**2.52**	**1.71**	**1.96**	**1.57**	**1.90**	**2.27**	**2.50**	**2.42**	**2.93**	**2.98**
**H905**	1.51	1.94	2.18	2.61	2.75	2.46	2.77	3.14	3.38	2.89
**H906**	**4.79**	**4.79**	**5.02**	**4.81**	**4.08**	**3.69**	**3.58**	**2.87**	**2.84**	**3.33**
**H907**	0.38	0.46	0.55	0.63	0.47	0.57	0.72	0.72	0.71	0.96
**H908**	0.50	0.68	0.65	0.73	0.76	0.95	0.98	1.08	1.07	1.23
**H91**	1.76	1.71	2.29	2.40	2.47	2.36	1.88	1.97	1.51	1.40
**H918**	0.00	0.00	0.00	0.00	0.00	0.00	0.00	0.09	0.53	0.70
**H919**	0.00	0.00	0.00	0.00	0.00	0.00	0.00	0.18	0.27	0.44
**tot**	100	100	100	100	100	100	100	100	100	100

**Table 4:** Children admitted to CAMHS with a hearing loss code (H90–H91) and with other associated codes.

Years	Patients with H.90–91 Diagnosis under UONPIA-ER Care	Patients with Only Hearing-Loss H.90–91	Patients with Hearing-Loss H.90–91 and Associated Diseases
**2011**	794	466 (58.7%)	328 (41.3%)
**2012**	876	521 (59.5%)	355 (40.5%)
**2013**	917	529 (57.6%)	388 (42.4%)
**2014**	957	524 (54.7%)	433 (45.3%)
**2015**	1053	561 (53.3%)	492 (46.7%)
**2016**	1058	558 (52.7%)	500 (47.3%)
**2017**	1118	565 (50.5%)	553 (49.5%)
**2018**	1114	550 (49.4%)	564 (50.6%)
**2019**	1125	474 (42.1%)	651 (57.9%)
**2020**	1140	467 (40.9%)	673 (59.1%)

**Table 5:** Other diagnostic codes present in the studied population and that are associated with hearing loss (percentage).

ICD-10 Diagnosis	2011	2012	2013	2014	2015	2016	2017	2018	2019	2020
**Q Q00–Q99 = Congenital Malformations. Deformations and Chromosomal Abnormalities**	13.9	15.02	15.46	15.89	16.87	17.05	16.97	17.97	16.16	17.18
**F70-F79 = Mental Retardation**	14.66	13.38	14.23	14.62	13.16	13.48	12.83	13.34	14.92	15.33
**P00-P96 = Certain Conditions Originating in the Perinatal Period**	8.08	7.97	7.63	7.61	7.82	7.82	7.43	7.05	5.88	5.44
**G G80-G83 Cerebral Palsy and other Paralytic Syndromes**	4.74	5.1	4.43	3.72	3.64	3.72	3.51	3.32	2.85	2.6
**F84 Pervasive Developmental Disorders**	1.51	1.33	0.94	0.85	1.63	1.71	1.96	2.14	1.92	2.41
**H3-H5 Visual Impairment**	5.28	5.52	5.56	5.83	5.5	5.29	5.75	5.81	5.08	5.13

**Table 6:** Incidence of genetic malformations and genetic syndromes per year.

Years	Q Q00–Q99 = Congenital Malformations. Deformations and Chromosomal Abnormalities	Prevalence Pop 0–17 Years:10000
**2011**	129	1.9
**2012**	147	2.1
**2013**	164	2.3
**2014**	188	2.6
**2015**	218	3.1
**2016**	229	3.2
**2017**	242	3.4
**2018**	260	3.7
**2019**	261	3.7
**2020**	278	4

## Data Availability

The IT management program, Sinpiaer, used by all the regional NPIA services across the Emilia Romagna Region (Italy), stores all of the data analyzed in this paper.
